# Artificial intelligence in psychiatry: clinical applications, limitations, and ethical challenges

**DOI:** 10.3389/fnbeh.2026.1864429

**Published:** 2026-07-23

**Authors:** Pedro Morgado

**Affiliations:** 1 Life and Health Sciences Research Institute (ICVS), School of Medicine, University of Minho, Braga, Portugal; 2 ICVS/3B’s-PT Government Associate Laboratory, Braga/Guimarães, Portugal; 3 Clinical Academic Center of Braga (2CA-Braga), Hospital de Braga, Braga, Portugal

**Keywords:** algorithmic bias, artificial intelligence, data governance, digital phenotyping, mental health ethics, psychiatry

## Abstract

Artificial intelligence (AI) is rapidly transforming psychiatric research and clinical practice, offering new capabilities in areas such as diagnosis, risk prediction, digital phenotyping, and treatment personalization. In the domain of diagnostic classification, machine learning models have demonstrated classification accuracy across major psychiatric disorders in internally validated research settings. In a distinct and non-equivalent domain, large language model–assisted clinical decision support has shown performance comparable to expert clinicians in a specific, structured benchmark task; this finding should not be generalized to open-ended clinical practice. However, this technological promise is shadowed by profound methodological, clinical, and ethical limitations. The majority of AI models in neuroimaging-based psychiatry carry a high risk of bias, external validation remains rare, and evidence of real-world clinical impact is scarce. Critically, the field is developing in a context where vast repositories of sensitive mental health data are increasingly controlled by large technology corporations. This trend raises urgent, yet underexplored, questions about data governance and commercial use, as well as broader concerns around accountability and long-term behavioral surveillance. Furthermore, the reliance of AI systems on statistical distributions to define normality risks encoding a historically unstable and culturally contingent concept as a medical standard, with particular consequences for the pathologization of human diversity. This perspective article argues that the psychiatric community must assume an active governance role, advocating for patient-centered data frameworks that do not reduce human suffering to a monetizable data stream.

## 1 Introduction

The integration of artificial intelligence into medicine has accelerated markedly over the past decade. Psychiatry, unsurprisingly, is no exception. From automated diagnostic classification to AI-assisted clinical interviews and smartphone-based passive monitoring, the scope of potential application is striking. A systematic review encompassing studies on diagnosis, monitoring, and intervention identified support vector machines, random forests, and AI-based conversational agents as the most frequently deployed approaches (Cruz-Gonzalez et al., 2025). Machine learning models have achieved classification accuracies exceeding 0.90 for anorexia nervosa, bulimia nervosa, and major depressive disorder (MDD). Furthermore, deep learning applied to nationwide register and genetic data has enabled cross-diagnostic predictions for schizophrenia, bipolar disorder, attention-deficit/hyperactivity disorder (ADHD), autism spectrum disorder (ASD), and MDD with AUC-ROC values between 0.71 and 0.82 (Allesøe et al., 2023; Zhang Z. et al., 2025; Zhang L. et al., 2025). These figures are technically noteworthy but must be interpreted within their methodological context. These metrics reflect performance in retrospective or internally validated research settings, and should not be interpreted as evidence of clinical readiness, external generalizability, or diagnostic validity in real-world psychiatric practice.

At the same time, a growing body of evidence points to important methodological weaknesses, an absence of external validation that is near-universal in certain domains [particularly passive sensing, where it is present in only 2% of studies (Shen et al., 2025)], and a fragmented and inconsistently enforced governance landscape that may expose patients - among the most vulnerable populations - to risks they cannot meaningfully consent to. A further, underappreciated risk is conceptual: AI diagnostic systems are built upon a notion of normality that has never withstood rigorous epistemological scrutiny (Catita et al., 2020; Fernandes et al., 2025). Importantly, when statistical averages derived from non-representative populations become the implicit reference for machine learning classifiers, pathologization of diversity becomes structurally likely - not merely possible - particularly when models employ diagnostic labels that conflate difference with disorder, or are deployed without cultural and functional contextualization.

When a machine-learning model predicts a suicide risk on the basis of a pattern it cannot explain, the traditional dyad of care is fundamentally challenged. While AI offers growing technical capability, it does so within a governance landscape that has yet to establish adequate and universally enforceable patient protections - and, for some commercial actors, within incentive structures that treat sensitive mental health data as a monetizable asset.

This perspective examines both the advances and the structural fragilities of AI in psychiatry, with particular attention to the ethical stakes of large-scale mental health data being held and processed by commercial entities whose primary accountability is to shareholders, not to patients and society.^1^

This article is a selective narrative perspective. Literature was identified through structured searches of PubMed, supplemented by citation tracking from recent systematic reviews and meta-analyses. Priority was given to systematic reviews, meta-analyses, and empirical studies, complemented by ethical, legal, and philosophical analyses where relevant. The selection reflects the author’s judgment about conceptual and clinical significance; no formal quality appraisal was applied. Readers should interpret the quantitative findings cited herein in light of the methodological limitations acknowledged throughout the text.

## 2 Current advances: where AI demonstrably adds value

The applications of AI in psychiatry span a broad and heterogeneous landscape. For clarity, they can be organized into four functionally distinct domains that differ substantially in their evidentiary base, clinical implications, and risk profile: (i) diagnostic and predictive classification models, including neuroimaging-based and register-based approaches; (ii) monitoring and digital phenotyping systems that capture passive behavioral signals; (iii) clinician-facing decision support tools, including LLM-assisted systems (where ‘frontier LLMs’ refers to large-scale generative language models at or near the current frontier of publicly available capability, including GPT-4 and successors, Claude, Gemini, and LLaMA-based systems); and (iv) patient-facing conversational agents. The evidence base, limitations, and ethical considerations discussed in this article are not uniformly applicable across these domains, and readers should bear these distinctions in mind throughout.

### 2.1 Diagnostic classification and risk prediction

Machine learning has shown promise for diagnostic classification in psychiatry, although results remain variable across settings. Importantly, the vast majority of included studies relied on internal validation, and the authors reported that external validation was rarely performed; calibration was not assessed in any study included in the neuroimaging review (Chen et al., 2023). These are critical methodological caveats: high AUC values derived from internally validated, retrospective datasets do not constitute evidence of clinical readiness, prospective generalizability, or diagnostic validity. AUC values provide limited information about calibration or clinical benefit; interpretation of model performance should be complemented by calibration analysis, assessment of sensitivity and specificity at the relevant decision threshold, and decision-analytic evaluation of net benefit.

A meta-analysis of 176 studies encompassing 60,926 participants reported an overall classification accuracy of 0.825 (95% CI: 0.810–0.839) for MDD using biological and neuropsychological markers (Zhang Z. et al., 2025). Models trained for eating disorders exhibited transdiagnostic generalizability, accurately classifying alcohol use disorder and MDD with AUC-ROC values between 0.75 and 0.93 — a finding consistent with shared latent risk architecture across psychiatric conditions (Zhang Z. et al., 2025). In neuroimaging, deep learning outperformed standard machine learning in 32 of 35 direct comparisons, with statistically significant advantages for ASD classification (Quaak et al., 2021). It bears emphasis that promising classification performance does not necessarily translate into diagnostic validity, clinical usefulness, or implementation readiness, and none of the studies reviewed in this subsection involved prospective clinical deployment.

### 2.2 Digital phenotyping and passive sensing

Smartphone-based passive sensing may represent one of the most ecologically valid approaches currently available in psychiatric assessment. Combining accelerometry, GPS patterns, social interaction proxies, sleep metrics, and screen-use logs, machine learning models have achieved classification accuracies exceeding 90% in some studies for anxiety detection (Shen et al., 2025). Passive sensing enables continuous and relatively unobtrusive monitoring, something that is difficult to achieve within the constraints of episodic clinic visits - a particularly important capability for conditions characterized by rapid mood fluctuation or subclinical prodromal states.

However, this promise must be set against substantial methodological limitations that have been consistently identified in systematic reviews of the field. Sample sizes in passive sensing studies are typically small - the median N in recent reviews is approximately 60 (Shen et al., 2025) - and follow-up periods rarely exceed 12 weeks. External validation is present in only 2% of studies (Shen et al., 2025). Missing data is a structural challenge: patterns of non-collection are themselves sociodemographically patterned, introducing bias that is rarely accounted for in published models. Device and platform dependence limits generalizability across operating systems and hardware generations. Furthermore, behavioral signals captured through passive sensing are not inherently psychiatric: the same mobility pattern may reflect different mental health states depending on employment status, socioeconomic context, or cultural norms (Adler et al., 2024). These contextual factors are particularly important: what constitutes “normal” mobility, sleep, or social interaction varies substantially across cultural, socioeconomic, and demographic groups, and models trained on predominantly urban, young, high-income-country populations cannot be assumed to generalize to other contexts. The inference from passive behavioral signals to psychopathology therefore requires caution, particularly given the near-universal absence of clinical validation in real-world deployment settings.

### 2.3 Clinician-facing decision support and patient-facing conversational agents

In a single structured evaluation using retrieval-augmented generation integrated with clinical guidelines, LLM-assisted decision support identified appropriate next-step treatments at rates comparable to expert clinicians in a specific benchmark setting, selecting poor treatment options less frequently than community clinicians for bipolar depression (Perlis et al., 2024). This finding is encouraging but should be contextualized: it reflects performance on a structured task rather than evidence of clinical equivalence in open-ended, real-world practice. Recent multi-task benchmarking of frontier LLMs in psychiatry has documented substantial gaps in clinical consistency and safety, particularly in multi-turn follow-up and management tasks, and current results support a cautious role for LLMs as aids to documentation or preliminary formulation rather than unsupervised decision-making in high-stakes clinical contexts (Fouda et al., 2026).

AI-powered conversational agents have been evaluated in a growing body of randomized trials. These trials constitute a distinct evidence domain from the classification and decision-support literature reviewed above, and the effect sizes reported here should not be read as corroborating evidence for the performance metrics described in section “2.1 Diagnostic classification and risk prediction.” Generative AI-assisted clinical interviews demonstrate higher agreement, sensitivity, and specificity compared to established rating scales when identifying self-reported diagnoses (Sikström et al., 2025). A meta-analysis of randomized evidence on AI-enabled personalized treatment planning reported reductions in anxiety (Cohen’s d = 0.62) and modest effects on depressive symptomatology (Hedges’ g = −0.35) (Yıldız and Yıldız, 2026). A complementary meta-analysis of 14 RCTs focused specifically on generative AI chatbots (*N* = 6,314) found a statistically significant but small aggregate effect (ES = 0.30; 95% CI 0.004, 0.59), with wide prediction intervals (−0.85 to 1.67) indicating substantial heterogeneity across interventions and populations (Zhang Q. et al., 2025). Evidence remains concentrated in the short term (most studies ≤ 12 weeks), and there is a notable lack of follow-up data, limiting conclusions about durability and safety of effect. These findings support the potential of conversational agents as accessible adjunctive tools, not as replacements for human therapeutic care.

## 3 Critical limitations: the gap between proof-of-concept and clinical reality

Despite these encouraging performance metrics, there are still several important limitations in the evidence base for AI in psychiatry, which substantially limit clinical applicability (Table 1).

**TABLE 1:** Summary of key methodological limitations in artificial intelligence (AI)-based psychiatric research.

Domain	Key finding	AI domain/evidence base	Clinical implication
Risk of bias	83.1% of models rated high overall risk (Chen et al., 2023)	Neuroimaging-based diagnostic models	Results cannot be assumed to generalize; clinical deployment premature
External validation	Present in only 2% of studies (Shen et al., 2025)	Passive sensing/digital phenotyping	Classification accuracy likely overestimated; performance in new populations unknown
Calibration	Absent in 100% of evaluated models (Chen et al., 2023)	Neuroimaging-based diagnostic models	Predicted probabilities cannot be interpreted clinically; risk of over- or under-treatment
Clinical applicability	None of the models rated as applicable to clinical practice (Chen et al., 2023)	Neuroimaging-based diagnostic models	Technical performance does not imply clinical utility or regulatory readiness
Temporal durability	Most interventions evaluated ≤ 12 weeks; long-term data absent (Abu-Mahfouz et al., 2026)	Conversational agents; passive sensing	Sustainability of clinical effects unknown; relapse or harm over longer horizons unstudied
Sample size	Median *N* = 60.5 in passive sensing (76% with *N* < 100); 71.7% of neuroimaging models with inadequate sample size (Chen et al., 2023; Abu-Mahfouz et al., 2026)	Neuroimaging classifiers; passive sensing	Overfitting likely; inter-individual generalizability not established
Subgroup/bias reporting	Rarely pre-specified or reported (Abu-Mahfouz et al., 2026); racial bias in treatment recommendations documented (Bouguettaya et al., 2025)	All AI domains; specifically LLM-based tools	Disparate performance across demographic groups may amplify existing health inequalities
Economic evaluation	Rare; integration, maintenance, and retraining costs not assessed (Abu-Mahfouz et al., 2026)	All AI domains	Implementation costs unknown; cost-effectiveness cannot currently be established

The scarcity of external validation is arguably one of the most consequential limitations. Internally validated AUCs typically cluster between 0.80 and 0.88, but prospective performance is almost never assessed (Abu-Mahfouz et al., 2026). Evidence remains concentrated in depression and anxiety, leaving schizophrenia, bipolar disorder, perinatal mental health, ASD in adults, and geriatric psychiatry substantially underrepresented (Jalali et al., 2025; Abu-Mahfouz et al., 2026). These gaps are critical since populations excluded from AI development are precisely those who may have most to gain from scalable, low-stigma support tools.

Beyond representation, there is a deeper conceptual limitation: the normative benchmarks against which AI classifiers define deviation are themselves drawn from historically specific populations, embedding an unexamined notion of normality - one that conflates statistical frequency with health - into the architecture of the model (Catita et al., 2020).

## 4 The ethical landscape: beyond algorithmic bias

### 4.1 Established concerns: bias, transparency, and accountability

Algorithmic bias in psychiatric AI is not hypothetical. When training data disproportionately represents certain demographic groups - which is common, given the concentration of research in high-income, predominantly White populations - models encode and amplify existing health disparities (Ostojic et al., 2024; Poudel et al., 2025). Subgroup performance analysis and prospective bias auditing are rarely pre-specified or reported (Abu-Mahfouz et al., 2026). The issue is compounded by the limited interpretability of many deep learning models: when a model recommends a treatment or flags suicide risk, clinicians cannot interrogate the reasoning, complicating both accountability and informed consent (Fisher, 2025; Giacobello, 2025).

The Integrated Ethical Approach for Computational Psychiatry (IEACP) framework - structured around beneficence, autonomy, justice, privacy, transparency, and scientific integrity - provides useful procedural scaffolding for navigating these challenges (Putica et al., 2025). Similarly, a consent framework has been proposed that evaluates AI use-cases according to model autonomy, departure from standards of practice, patient-facing nature, clinical risk, and administrative burden (Rose and Shapiro, 2024). These frameworks are valuable, but their adoption remains voluntary and their enforcement non-existent.

### 4.2 AI, normality, and the pathologisation of diversity

Central to the ethical critique of AI in psychiatry is a problem that predates the technology itself: the concept of normality. In medicine, “normality” has never acquired a stable, universal definition. It has functioned variously as a statistical descriptor, a cultural ideal, a functional standard, and a value judgment - often simultaneously (Boorse, 2014; Wakefield, 2014; Catita et al., 2020). A critical analysis of successive editions of the DSM reveals that normality-related terminology has increased from eight mentions in DSM-I to 198 in DSM-5, yet the term remains ambiguous, context-dependent, and epistemologically undefended (Fernandes et al., 2025). When the DSM describes a “normal-looking body” or “normal social communication,” it encodes cultural majority expectations as medical criteria - a sleight of hand that dimensional psychiatry has yet to fully confront.

AI systems inherit and amplify this problem. Machine learning classifiers are trained on datasets that instantiate a particular historical, geographical, and cultural distribution of behavior. The decision boundary between “normal” and “abnormal” is therefore not derived from a neutral, universal standard - it is derived from whatever population happened to be represented in the training data. For populations outside that distribution - whether by culture, neurodivergence, economic circumstance, or gender expression - the model’s output is not a clinical judgment but a measure of distance from an unexamined majority norm. It is important to distinguish, analytically, between statistical atypicality, diagnostic classification, clinically significant distress, functional impairment, cultural difference, disability, and neurodivergence: none of these concepts can substitute for another in defining the threshold at which AI-assisted assessment crosses from pattern recognition into clinical classification (den Houting, 2019; Chapman, 2020; Kirmayer et al., 2024).

Digital phenotyping exacerbates this dynamic. Continuous behavioral monitoring captures deviations from statistical baselines in mobility, sleep, social interaction, and communication patterns. These baselines are themselves constructed from populations that are predominantly young, urban, connected, and drawn from high-income countries. Equating statistical average with mental health, as Fernandes et al. (2025) warn, does not resolve the definitional problem of normality - it conceals it behind a veneer of quantitative precision. The result is a system that may be highly accurate at identifying difference, while remaining silent on whether difference constitutes disorder.

Pathologisation becomes structurally likely - rather than merely possible - when models are trained on unrepresentative populations, employ diagnostic labels that conflate difference with disorder, or are deployed without cultural and functional contextualization. The risk is not inherent to the use of statistical methods per se, but to the specific choices made in training data selection, label design, threshold setting, and clinical workflow integration (Kirmayer et al., 2024; Kirmayer, 2025).

These concerns are not merely theoretical. Empirical evidence of differential AI performance across demographic groups is accumulating. A recent study systematically varied racial identity across psychiatric case vignettes presented to four leading LLMs (Claude, ChatGPT, Gemini, NewMes-15) and found that models consistently proposed inferior treatment recommendations when patient race was explicitly or implicitly indicated as African American - while diagnostic decisions showed comparatively little variation (Bouguettaya et al., 2025). Similar findings have been documented in broader mental health NLP evaluations, where nationality and religious identity were associated with increased model error in predicting mental health diagnoses. Machine learning models that optimize aggregate performance across an entire population systematically smooth out or obscure race-related and clinically meaningful subgroup variance - a failure that is particularly consequential in psychiatry, where the cumulative effects of racism-related stress produce distinct physiological and psychological signatures. Subgroup performance auditing is rarely pre-specified or reported, meaning that disparate error rates often go undetected until tools are in widespread use.

This has direct implications for neurodivergent populations, cultural minorities, and individuals whose life circumstances produce behavioral patterns systematically divergent from training data norms - not because they are unwell, but because they are different. An AI psychiatry that cannot distinguish between pathological deviation and human diversity is not merely imprecise: it is unjust. Acknowledging this requires more than philosophical caution: it requires statistical deviation, functional impairment, clinical disorder, and cultural difference to be treated as analytically distinct categories - none of which can substitute for another in defining the threshold at which AI-assisted assessment crosses from pattern recognition into clinical classification.

### 4.3 The corporate data question: an under addressed structural risk

A dimension that receives insufficient attention in the psychiatric AI literature is the structural relationship between mental health data and commercial technology entities. It is important to distinguish among the different actors involved, as they carry materially different legal obligations, access rights, and commercial incentives: data controllers (typically the clinical institution or consumer application), data processors (cloud infrastructure providers, foundation model developers), and third parties (advertisers, insurers, and other downstream recipients).

Digital phenotyping and passive sensing generate extraordinarily sensitive behavioral data - moment-to-moment mobility, sleep, social interaction, communication content - that is collected continuously and processed on infrastructure owned by private corporations. Only 14% of studies in a recent scoping review addressed data anonymisation (Shen et al., 2025), and economic evaluations accounting for integration, maintenance, or data-retraining costs are rare (Abu-Mahfouz et al., 2026).

It is also important to distinguish between identifiable, pseudonymized, and inferred data. Inferred outputs - such as a calculated suicide risk score or a predicted diagnostic category - can carry significant governance implications even when the underlying input data is pseudonymized, since such outputs may be re-identifiable or actionable in contexts (insurance, employment, civil proceedings) where the original data would not be disclosed.

The implications extend well beyond current research contexts. Mental health data, once collected, does not automatically disappear: data governance frameworks vary substantially across jurisdictions and commercial actors, and documented examples of mental health apps sharing user conversations and clinical assessments with third-party advertisers without clear consent have already emerged (Botes, 2025).

In the United States, legal analysis of 50-state regulation identifies fragmented oversight, with state legislatures serving as de facto regulatory laboratories while federal authority remains absent (Shumate et al., 2025). In contrast, the European Union has established a more structured - though still imperfectly enforced - regulatory environment. The GDPR mandates data minimisation, purpose limitation, and explicit consent for sensitive health data processing; the EU Artificial Intelligence Act establishes a risk-based classification framework under which certain AI systems used in clinical decision-making and risk assessment in mental health contexts may qualify as high-risk, imposing transparency, accuracy, and human oversight requirements; and the forthcoming European Health Data Space seeks to regulate the secondary use of health data across member states. These frameworks do not resolve all of the concerns raised in this article, but their existence means that the governance landscape cannot accurately be described as uniformly absent. The more accurate characterization is one of fragmented and inconsistently enforced governance, with substantial variation across jurisdictions and actors (European Parliament and Council of the European Union, 2016, 2024; European Commission, 2022).

The risks of data exposure extend to insurance and employment contexts, although quantifying the frequency with which this occurs remains methodologically challenging. The probabilistic nature of large language models - and the documented tendency to produce clinically plausible but factually inaccurate outputs - makes downstream harm difficult to predict and harder to attribute (Perlis, 2026).

There is a fundamental asymmetry at stake: patients often contribute data during periods of psychological distress, when their ability to assess long-term consequences may be limited. Meanwhile, corporations retain access to what becomes a long-term and potentially valuable data asset. This asymmetry may not simply be a side effect of AI development in psychiatry; for some commercial actors, it could represent a central driver. Mental health is a vast, underserved market, and behavioral data is the raw material through which it is to be exploited.

This is not an argument against technological progress, but a recognition that the entities building these tools are driven by structural incentives that may conflict with patient welfare, and that the psychiatric profession must not outsource its ethical responsibilities to terms-of-service agreements or corporate ethics boards.

Addressing these structural risks requires governance frameworks with enforceable substance rather than voluntary commitments. At minimum, this includes: data minimization (collecting only what a defined clinical function requires); purpose limitation (prohibiting secondary use, including model retraining, without explicit continuing consent); independent oversight of commercial mental health data practices; restrictions on sharing with third parties, including insurers and employers; structured post-deployment auditing; and transparent public disclosure of data retention and deletion policies. Patients who consent to data collection during acute episodes of illness should not be permanently bound by decisions made at their most cognitively compromised.

### 4.4 The therapeutic relationship in triadic space

The integration of AI reconfigures the therapeutic relationship from a dyadic exchange - clinician and patient - into a triadic one, with AI as a third, non-transparent participant (Giacobello, 2025). Patients report willingness to share anonymized data for research purposes but express explicit concern about data reaching insurance or technology companies (Vo et al., 2023). Both patients and clinicians doubt AI’s capacity for empathic care and reject proposals to replace human clinical relationships with AI-mediated ones (Vo et al., 2023). These preferences deserve structural weight, not merely rhetorical acknowledgment.

What distinguishes AI from other third-party presences in the clinical encounter is its invisibility. A human observer - a trainee, a supervisor - can be named and incorporated into the negotiation of trust. AI cannot. When a patient speaks in a setting equipped with AI-assisted transcription, passive sensing, or algorithmic risk monitoring, the third presence is structurally operative yet imperceptible. This AI agent records, classifies, and makes inferences from the silences, hesitations, and affective registers that constitute the core material of psychotherapeutic work.

Confidentiality and the assurance that what is disclosed goes no further are not procedural formalities - they are the conditions under which patients risk saying what they have not yet been able to say elsewhere. Their disruption, even by an invisible and inaudible agent, alters the therapeutic frame in ways that are clinically consequential. Patients who know or suspect that their words or biometric data are being processed beyond the dyadic encounter may self-censor or withhold precisely the most clinically significant material - an effect empirically documented in contexts of continuous digital monitoring (Büchi et al., 2022; Rogan et al., 2024). When adolescent patients were consulted about the introduction of passive sensing into their care, their primary concern was not technical but relational: who has access, for how long, and to what end (Sonig et al., 2024). The frame is compromised not by what the AI reveals, but by what its presence forecloses.

Automation bias - the tendency for clinicians to defer uncritically to algorithmic outputs - represents a related concern. Where AI tools become embedded in routine workflows, the conditions for meaningful human oversight erode. The risk of diminished clinical skill over time, through reduced practice of the very judgments AI is intended to support, has been raised but remains empirically unstudied (Perlis, 2026).

Translating these concerns into clinical practice requires specific structural commitments. Disclosure of AI involvement should be treated as a component of informed consent: patients have both a right and a clinical interest in knowing when AI-assisted transcription, algorithmic risk scoring, or passive monitoring is operative within their care (Park, 2024). It is important to distinguish between therapeutic alliance (a measurable relational construct), perceived empathy (a user-reported subjective experience that may be influenced by anthropomorphic design), and the moral and emotional agency that current AI systems do not possess. Anthropomorphic design may increase engagement and alliance scores without resolving the ontological gap between simulated and genuine relational presence.

Consent should be treated as a dynamic process - not a one-time signature - particularly for tools that collect data continuously or that may be updated or repurposed over the course of care. Limits on fully autonomous patient-facing systems in high-risk clinical contexts (crisis triage, medication initiation, risk assessment) should be specified in institutional policy rather than left to individual clinician discretion. Monitoring for automation bias should be built into the routine quality review of AI-assisted clinical tools, including tracking of rates at which clinicians override algorithmic recommendations and the outcomes of those overrides. The atrophy of clinical judgment through over-reliance on algorithmic outputs is not a speculative future risk - it is a plausible consequence of any workflow in which AI recommendations become the default starting point for clinical decision-making.

## 5 Discussion

### 5.1 Limitations of this perspective

This perspective has several limitations that readers should bear in mind. The literature selection was not systematic, and no formal quality appraisal was applied to included studies. Evidence is predominantly drawn from English-language publications originating in high-income countries, which limits the generalizability of the conclusions to other linguistic and economic contexts. The evidence base for large language models in clinical psychiatry is evolving rapidly, and some conclusions regarding their capabilities and limitations may require revision as new studies emerge. The legal and governance analysis is selective: it prioritizes the United States, South Africa, and the European Union, and a comprehensive comparative legal analysis across jurisdictions was beyond the scope of this article. These limitations do not invalidate the central arguments, but they are relevant to how the evidence and recommendations should be interpreted.

### 5.2 A field at an inflection point

Artificial intelligence in psychiatry is at a pivotal point. While AI’s technical prowess surges, our governance frameworks remain stagnant. The tools are outpacing the frameworks designed to govern them. The field risks repeating the pattern of other digital health innovations: enthusiastic proof-of-concept research, premature clinical deployment, and belated discovery of harm - with those harms falling disproportionately on the patients least equipped to seek redress.

The path forward requires, at minimum, three commitments that the field has so far resisted making routine: first, prospective, multi-site validation studies with diverse samples must become the expected standard rather than the exceptional achievement; second, standardized reporting frameworks must be enforced by journals and funding bodies rather than left to individual discretion - several AI-specific frameworks already exist for this purpose: TRIPOD+AI for transparent reporting of prediction model studies, CONSORT-AI and SPIRIT-AI for clinical trials and protocols involving AI interventions, DECIDE-AI for early-stage clinical evaluation of AI-based decision-support systems, and FUTURE-AI for trustworthy and deployable AI in clinical settings (Cruz Rivera et al., 2020; Liu et al., 2020; Vasey et al., 2022; Collins et al., 2024; Lekadir et al., 2025); and third, economic evaluations must account for implementation costs, data governance, and post-deployment monitoring. Presenting performance metrics in a cost-free vacuum is no longer defensible.

Concretely, the field should commit to the following minimum standards: (1) Prospective, multi-site external validation with demographically diverse samples, reported with calibration metrics and subgroup analyses, should be required before any AI diagnostic or predictive tool is presented as clinically ready. (2) Adherence to established AI-specific reporting frameworks (TRIPOD+AI, CONSORT-AI, SPIRIT-AI, DECIDE-AI, FUTURE-AI, as appropriate to study design) should be required by journals and funding bodies. (3) Economic evaluations must account for implementation costs, data governance infrastructure, and post-deployment monitoring, not only technical performance. (4) Subgroup bias audits, including at minimum analysis by age, sex, gender, race/ethnicity, and socioeconomic status, should be pre-registered and publicly reported. (5) Continuing consent models should be adopted for any tool involving ongoing data collection, with provisions for withdrawal and data deletion. (6) Human oversight requirements should be specified in regulation for high-risk clinical AI functions, with clear accountability when algorithmic recommendations contribute to patient harm. (7) Post-deployment safety monitoring - including adverse event reporting and regular re-validation as patient populations and clinical contexts change - should be treated as a regulatory requirement.

### 5.3 Data sovereignty as a clinical imperative

Beyond methodological reform, the psychiatric community must engage substantively with the question of data sovereignty. Mental health data is not like other health data: it is deeply intimate, it carries significant stigma, and its misuse can have life-altering consequences for employment, insurance, and civil rights. The collection of this data at scale - through apps, wearables, and AI-assisted clinical encounters - without robust governance is not only an individual privacy concern, but a structural risk.

What governance requires, at minimum, is a serious reckoning with how mental health data moves through systems not designed with patients in mind. Data must not travel beyond its stated purpose, tools must collect no more than their function requires, and consent must be treated as a continuing process. A patient who agrees to data collection during an acute episode is not the same patient who will live with the consequences of that decision years later. Governance frameworks that ignore this are not neutral but a choice made on the patient’s behalf without their meaningful participation.

### 5.4 The irreplaceable human dimension

The future of psychiatry will not be determined by the precision of our instruments, but by who owns them. AI offers real and growing value in psychiatry: standardizing assessments, detecting signals too subtle or high-dimensional for clinical intuition, extending the reach of support to populations otherwise without access. These contributions are not trivial, and dismissing them in the name of precaution would itself cause harm. But the introduction of these tools must not be permitted to displace human clinical relationships, reduce staffing in mental health services, or transfer the locus of expertise from trained clinicians to algorithmic systems that cannot be held accountable, cannot feel empathy, and cannot adapt to the irreducible particularity of a human life in distress.

In practice, AI is likely to function best as a support tool within clinical decision-making, rather than as a replacement for clinician judgment in psychiatry.

This distinction becomes most acute in the case of conversational agents and chatbots, which are increasingly designed to simulate the affective texture of human interaction - adopting warm tone, responsive cadence, and the appearance of empathic attunement. Some systems are deliberately rendered humanoid in voice, avatar, or relational style, precisely because anthropomorphic design increases engagement, perceived trust, and scores on therapeutic alliance measures - though it is important to distinguish between measured alliance (a relational construct), user-reported perceived empathy (a subjective response to design), and the moral and emotional agency that current AI systems categorically do not possess (Vo et al., 2023). Yet no degree of design sophistication resolves the fundamental ontological gap: a chatbot that appears to listen is not listening; one that appears to care is not caring. However, hyperrealistic the interface, the entity on the other side of the exchange has no interiority, no stake in the patient’s life, and no capacity to be affected by what it is told. This matters clinically because psychotherapy does not operate through the simulation of human relationship - it operates through human relationship itself.

The therapeutic encounter is not merely an information exchange that could, in principle, be optimized by a sufficiently advanced language model. It is a meeting between two beings, both capable of vulnerability. Change does not emerge from data processing; it emerges from the irreducible reality of that shared human presence. Designing systems to appear human while being categorically non-human is not a neutral technical choice. Rather, it is a form of deliberate ambiguity that exploits the patient’s relational needs without the ability to truly meet them. This practice risks substituting the appearance of care for its substance at the precise moment when the distinction matters most.

Psychiatry has always been, at its core, the discipline that refuses to reduce human suffering to a mechanism - and it is precisely that refusal that must now be brought, with clarity and conviction, to bear on the tools it chooses to adopt. What AI cannot replicate - and what psychiatry must not surrender - is the irreducible act of one human being choosing to remain present with another in their suffering: not processing it, not predicting it, but witnessing it.

## Data Availability

The original contributions presented in this study are included in this article/supplementary material, further inquiries can be directed to the corresponding author.

## Footnotes

1 Throughout this article, the following distinctions are maintained. *Classification* refers to the assignment by a model of an instance to a diagnostic category. *Prediction* refers to the estimation of a future clinical outcome (e.g., relapse risk). *Clinical decision support* refers to AI-generated information or recommendations provided to a clinician within a clinical workflow. *Treatment recommendation* refers specifically to a suggested therapeutic action generated by an AI system. *Digital phenotyping* refers to the collection and analysis of passively acquired behavioural data from personal devices. *Conversational agents* (also: chatbots, virtual agents) refer to software systems designed to interact with users through natural language, whether rule-based, retrieval-based, or generative.
